# Solila

**Published:** 1896-06

**Authors:** Theodore Frick

**Affiliations:** Switzerland


### Translations.

### SOLILA.[1]

BY THEODORE FRICK, D.D.S., SWITZERLAND.

If we glance through the professional periodicals of the day, we find that their advertising pages offer more different materials for filling than ever before. And many, nay most, of these preparations are equipped, on paper, with such superior qualities and such promising names that one might almost believe that operative dentistry had reached the acme of perfection, and that artificial teeth were no longer necessary, except for the aged. However, the more such aggressive claims multiply, the more difficult does it become for the individual to distinguish between good and bad. Many may have grown so sceptical by reason of the disappointments that they have experienced as to try no further novelties. So much

[1] Translated from the Schweizerische Vierteljahrsschrift für Zahnheilkunde, Marz, 1896.

the more is the duty of those who have tested new preparations thoroughly, and by trustworthy methods, to make their opinion publicly known to a larger circle, whether the result of their tests has been favorable or unfavorable.

For many decades, gold has properly been rated as the most enduring material for fillings. The principal reason why there has been a continual effort to substitute other materials for it is not, as is frequently claimed, the high price of the material itself, but the fact that its use demands so much time, skill, labor, and effort on the part of the operator. Or, in other words, it is not "dear gold" that makes gold fillings dear, but the time and the manual facility required to produce them. Now, the most recent of the gold preparations, called "solila," spares us a part of these two exactions, and, for this reason, deserves to be raised above the level of ordinary "novelties."

"Solila," a crystal gold, is prepared by its inventor, Dr. E. de Trey, a Swiss dentist, and is to be had at all dental depots. Its treatment is essentially different from that of all other gold preparations. The operator who intends to work with solila should, if possible, secure an opportunity of witnessing a practical demonstration of its use. As I have already inserted about eight hundred gold fillings in the mouth, using for the purpose about ten ounces of "solila," I consider that I have sufficient experience to offer a few practical suggestions. I assume that the reader has already made good gold fillings with the preparations of gold heretofore in use, and therefore I call attention only to those points in which there is deviation from present practice.

Even in the preparation of the cavity we have a slight difference. We can almost entirely dispense with anchorages and under-cuts. It is a matter of course that this gold, like any other, does not adhere to the walls of the cavity, but remains firmly fixed in the excavation only by reason of its form. After the caries has been removed, and *the edges of the enamel have been sufficiently cut down* and polished, care should be taken, if possible, that the interior of the excavation be somewhat larger than the orifice. If this is impracticable on account of the proximity of the pulp, or for other reasons, *very slight* under-cuts are made, not deeper than a fissure bar will leave in the dentine. We are naturally guided in this by the general rule, that the more pronounced the contour of a filling is, the more thorough should be its anchorage. Nevertheless, deep under-cuts and drilled anchorages are to be altogether avoided, because, as we shall see later on, it would only occasion a useless waste of time to fill them. Excavations of any kind, to be used as starting-points, are wholly unnecessary.

Coming now to the process of filling, we will consider first the gold itself. "Solila" is put on the market in small boxes, containing one-eighth ounce each. It is prepared in four different numbers, of which each has its own peculiar properties and corresponding use. Nevertheless, it is possible, in case of necessity, to produce a good gold filling with one of the numbers only. Nos. 1 and 2 are very soft and almost plastic; they will probably be used most frequently. They are especially adapted for beginning a filling and for continuing it up to the point where, with a concave surface, it reaches the edge of the enamel. No. 1 is thicker and denser and is consequently calculated for large cavities; No. 2 is somewhat thinner for smaller cavities and excavations. No. 4 is rather more rigid and denser than the other three numbers; it is of service principally for the construction of a good surface and for the building up of contours. No. 3 is a very thin and extremely pliable gold, particularly available for the entire filling in very small cavities that are difficult of access. It is also suitable for finishing surfaces and contours in place of No. 4, when the latter would involve too large an application. In the small boxes, the gold is arranged in narrow strips, separated by soft cushions. This peculiar method of packing is, in itself, an indication that solila should be handled as gently as possible before insertion, and that it should never be subjected to pressure. It must not be touched, except with the pliers, preferably with the gold-pliers made especially for this purpose, which, by reason of their slender tongs, make any compression of the gold taken up almost impossible. For cutting the gold into smaller pieces, it is best to use the De Trey shears, which avoid flattening the gold along the edges of the cut. The pliers should also be used for introducing the separate pieces into the cavity; "picking up" with the plugger, as in the use of gold cylinders, is impossible.

Solila gold is a pronounced cohesive gold, and, as such, is heated before insertion. This can be done directly over a clear flame, or, better still, on a piece of mica. The thicker numbers must be heated on both sides, and must, therefore, be turned. If overheated, the gold becomes rigid and less pliable.

For filling, the inventor of "solila" has manufactured special instruments. These have strong, thick handles, very much like the old-fashioned soft-gold pluggers. The extremities of these instruments are considerably larger than those of former gold pluggers, and have convex surfaces only. They are spherical, hemispherical, and thumb-shaped, and some of them have the form of an inverted cone. There are slight serrations, not only upon the extreme end, but also upon the sides of the plugger, the principal purpose of which is to avoid any slipping of the instrument. It is best to use hand-pressure in inserting the gold. While it was customary, in the employment of gold-foil and cylinders, to execute a sharp pushing movement in a definite direction, and so to imitate the blow of a hammer, the movements required by the solila pluggers are more of a rotary, rocking, or rolling character. Accordingly, the operator will often grasp the plugger with the entire hand, and will thus be enabled to exercise a strong pressure with relatively slight exertion.

In beginning to fill a cavity, a piece of gold (No. 1 or 2), of about the size of the opening, is introduced with the pliers, pressed quite lightly with as large a plugger as possible, until it is firmly fixed in the bottom of the cavity, and then condensed, the instrument being rolled and rocked back and forth with strong pressure. It will at once be noticed, in doing this, how plastic the gold is, how closely it adapts itself to the walls of the cavity, and how it gives steadily in a lateral direction, without any tendency to roll or loosen. It is quickly condensed; even at the second or third pressure given it, the operator will feel a fully condensed mass under the instrument. The process is continued in the same way, laying on piece after piece and condensing. The pieces are very dense; a piece of the size of an ordinary gold cylinder represents about six times its quantity of gold. It is, therefore, customary to work with pluggers as large as possible, but the operator should not omit to go over the surface from time to time with fine instruments, employing equally strong pressure. If it is possible to make more marked depressions with these, such depressions should be filled up at once (preferably with No. 3), before proceeding with the coarser instruments. The movements in condensing should always be made from the centre of the cavity towards the edges; the latter should receive especial attention, and care should at all times be taken, that the gold may present a concave, never a convex surface. The old principle, applied to the soft-gold fillings, according to which the operator began at one wall of the cavity, then covered the other, and finally forced the last pieces into place in the centre, has, therefore, been wholly abandoned. We simply begin at the bottom and continue to work throughout the entire width of the cavity, until we reach the edges of the enamel. We then proceed, using No. 4 or No. 3, in the manner described above, until the surface has the form desired. Even at this last stage, the operator should not fail to go over the ground with fine instruments and fill up any depression made in this way with small pieces of gold. The filling is finished precisely as in the case of gold fillings generally; it will be observed in this connection that the surface is very hard, and that working it down is a very slow process. Care should, therefore be taken, not to apply too much at any point in building up the contour. By reason of its even density, solila gold quickly becomes heated under friction, a fact which should be borne in mind, when using disks and other dry polishing instruments. The surface is capable of taking a very fine polish.

These are the suggestions which I am able to give with reference to the use of solila. So far as its qualities of endurance are concerned, it is true that we cannot, at the present time, allow ourselves any absolutely conclusive judgment; but there is not the slightest apparent reason to doubt them in any way.

In conclusion, I most earnestly recommend the use of solila gold. Those who have not succeeded heretofore in making permanent gold fillings, will likewise have small success with solila, and I warn every one against its use who believes that good gold fillings can be made with this preparation in unskilled hands without labor, without close attention, or without thoroughly preparing the cavity. But, on the other hand, I am convinced that any one, who has been working carefully and successfully with the previous preparations of gold, will make at least equally good fillings with this new gold in shorter time and with greater certainty, and that he will gradually venture with success upon complicated and difficult tasks that he would not have undertaken before.

